# Acquire: an open-source comprehensive cancer biobanking system

**DOI:** 10.1093/bioinformatics/btv012

**Published:** 2015-01-07

**Authors:** Heidi Dowst, Benjamin Pew, Chris Watkins, Apollo McOwiti, Jonathan Barney, Shijing Qu, Lauren B. Becnel

**Affiliations:** ^1^Dan L. Duncan Cancer Center, ^2^Department of Medicine, Section of Hematology and Oncology and ^3^Department of Molecular and Cellular Biology, Baylor College of Medicine, 1 Baylor Plaza, Houston, TX 77030, USA

## Abstract

**Motivation:** The probability of effective treatment of cancer with a targeted therapeutic can be improved for patients with defined genotypes containing actionable mutations. To this end, many human cancer biobanks are integrating more tightly with genomic sequencing facilities and with those creating and maintaining patient-derived xenografts (PDX) and cell lines to provide renewable resources for translational research.

**Results:** To support the complex data management needs and workflows of several such biobanks, we developed Acquire. It is a robust, secure, web-based, database-backed open-source system that supports all major needs of a modern cancer biobank. Its modules allow for i) up-to-the-minute ‘scoreboard’ and graphical reporting of collections; ii) end user roles and permissions; iii) specimen inventory through caTissue Suite; iv) shipping forms for distribution of specimens to pathology, genomic analysis and PDX/cell line creation facilities; v) robust *ad hoc* querying; vi) molecular and cellular quality control metrics to track specimens’ progress and quality; vii) public researcher request; viii) resource allocation committee distribution request review and oversight and ix) linkage to available derivatives of specimen.

**Availability and Implementation:** Acquire implements standard controlled vocabularies, ontologies and objects from the NCI, CDISC and others. Here we describe the functionality of the system, its technological stack and the processes it supports. A test version Acquire is available at https://tcrbacquire-stg.research.bcm.edu; software is available in https://github.com/BCM-DLDCC/Acquire; and UML models, data and workflow diagrams, behavioral specifications and other documents are available at https://github.com/BCM-DLDCC/Acquire/tree/master/supplementaryMaterials.

**Contact:** becnel@bcm.edu

## 1 Introduction

Access to high quality human cancer and matched normal tissues for ‘omics experimentation is critical for developing a better understanding of these diseases to design improved diagnostics and therapeutics. Human tissue biorepositories, or biobanks, can provide tissues and associated participant, pathology and specimen data to the researchers who collected them and/or the scientific public (Ayers *et al.*, 2007; Barnes *et al.*, 2013; Chen *et al.*, 2011; Palmer, 2007). Biobanking standards provide guidance and best practices on how to collect, store and manage tissues to better assure high-quality DNA, RNA, protein or other derivatives such as cell lines and patient-derived xenografts (PDX) for research (e.g. International Society for Biological and Environmental Biorepositories [ISBER] 2012 Best Practices, http://bit.ly/1znXuEU). As such many commercial and some open-source software solutions exist to manage daily repository operations and the inventory of a biobank. These products may not cover all aspects of biobanking operations outside of the repository, such as derivative creation, specimen annotations, quality control (QC) mechanisms or resource distribution. Some commercial systems can be prohibitively expensive for biobanks with limited financial resources. Two popular open-source options, caTissue Suite and OpenSpecimen, which is an optimized version of the caTissue codebase, have robust inventory support. Their scope lacks some features for other important operational aspects of a biobank, such as QC or oversight committee reviews of tissue requests.

Applications for use in any type of biobank may not have out-of-the-box support for standard data elements and code sets appropriate for cancers, which can reduce adoption of standard vocabularies and data elements. The ability to dynamically add data elements in electronic forms is a popular solution to capture custom data. If these data entry forms are poorly designed, however, issues with data interchange, standards compliance and getting data back out of the system can arise. Further, many tools were not created to support a newer case for biobanks embedded within an ‘omics context, which can range from collaborating research laboratories or centers generating genomic or proteomic data to Clinical Laboratory Improvement Amendments (CLIA)-certified diagnostic laboratories. For example, correctly modelling biomarkers, which can be ‘patient level’ such as genomic information on germline variants; ‘case level’ with somatic variants within tumors; or even ‘aliquot level’ for tests of protein expression or activation within a slide, is complex and requires understanding of the biomedical domain along with data and object modelling best practices.

Like many biomedical informatics groups, we were tasked with supporting the informatics needs a variety of cancer biobanks that vary in degree of centralization. These include national and Texas-statewide federated banks such as the Texas Cancer Research Biobank, local repositories of national banks such as the AIDS and Cancer Specimen Resource, local Specialized Programs of Research Excellence, core facilities and individual investigators’ collections. Each of these groups had sufficiently similar needs that a central platform could support them all. Many of these biobanks had modest informatics support budgets and could not afford expensive software solutions. All had shared needs for robust inventory management, data QC/QA (quality assurance), tracking of specimen requests and distributions, resource allocation committee support (RACs), etc. In addition, many of these biobanks managed specimens collected as part of clinical trials protocols, so any informatics system would need to be able to interoperate with a clinical trials management system. After carefully reviewing available commercial-off-the-shelf and open-source options in 2011, we determined that at that time none fully met the requirements of these biobanks.

To this end, we developed Acquire to manage the full lifecycle of a specimen and its derivatives, distributions in different contexts (e.g. to xenograft creation or genomic analysis cores) and requests. Although Acquire supports dozens of biobanking workflows in various facets of the lifecycle, it is not a monolithic system. Rather, it is composed of several modules of software to facilitate updates and even wholesale replacement of modules should newer software better meet business needs at a lower cost in the future. A test instance of Acquire that has been loaded with masked data is available at https://tcrbacquire-stg.research.bcm.edu, login: reviewer1@review.com, password: Change#1. The codebase, all models, deployment guides and customization notes for Acquire are freely available at https://github.com/BCM-DLDCC/Acquire.

## 2 Methods

### 2.1 Application and databases

Acquire (current version 2.3.1) is a modular Java Enterprise Edition 6, web-based application built around an Oracle 11 g database for structured data and a MongoDB store for image, pathology report or other file uploads. Through the use of Hibernate, Oracle can be swapped for other relational databases. The application runs in JBoss 7 on Red Hat Enterprise Linux, though other Unix-based operating systems (e.g. CentOS) may be utilized. Acquire is a modular system; caTissue Suite 1.2 was adopted as the biobank inventory module, whereas all other modules were constructed by the authors. caTissue technical specifications are described within its online deployment guide (http://1.usa.gov/1vRKzWO). For non-caTissue modules, the system uses the model-view-controller architecture to manage its various components. The model component of the system uses Java Persistence API (JPA) for Entity persistence. The views (i.e. the webpages) are rendered via a combination of AJAX technologies and core JSF and Primefaces components for the user interface. Acquire associates universally unique identifiers with each participant and specimen, aliquot or derivative. Security is maintained through restricting connections only to HTTPS connections and applying secure socket layer certificates to the application host.

### 2.2 Security

To provide a seamless end-user experience for workflows that span two or more Acquire modules, central authentication service (CAS) was utilized for single sign-on to seamlessly integrate authentication and authorization across Acquire’s modules. CAS supports end users credentialed at the institution that hosts Acquire and roles. Users are assigned permission-based roles for one or more biobank programs or individual sites underneath a given biobank program. General roles include: public scientist, non-PHI (protected health information) technician, PHI viewing supervisor, RAC member and RAC coordinator. Further granularity is added by individually adding module permissions to each account to allow for users who fulfill multiple roles within the system depending on the program. CAS ties into hosting institutional active directory (AD) LDAP for authentication and secondarily authenticates within Acquire’s local Oracle database. All local passwords are stored in a salted hash.

### 2.3 UML models and documentation

The Acquire logical model was created using Enterprise Architect. End-user documentation, README files for deployment and a deployment guide were created in Microsoft Word or similar word processing programs. All documentation is available at https://github.com/BCM-DLDCC/Acquire/tree/master/supplementaryMaterials/.

### 2.4 Web browser validation

Acquire has been optimized for Firefox 32+, Chrome 37+, Safari 7+ and Internet Explorer 11+ with validations performed in BrowserStack.

## 3 Results

Though banking protocols and processes differ slightly among institutions, all must have processes for identifying potential subjects and obtaining consent, collecting specimens and capturing data. Acquire supports the full biobanking lifecycle for specimen and data collection including:

- *Participant data capture*: consent, demographics and clinical annotations
- *Specimen data capture*: collection, accession, storage, maintenance, aliquoting or derivative creation and pathologic findings
- *Data capture workflow management*: status checks on which key participant and specimen data are incomplete
- *Data quality checks*: reports to help identify data or specimen collection quality problems
- *Resource allocation*: public researcher specimen requests, RAC request reviews and specimen distributions
- *Reporting*: data mining for biobank personnel and restricted-access searching for public researchers

**Fig. 1.**
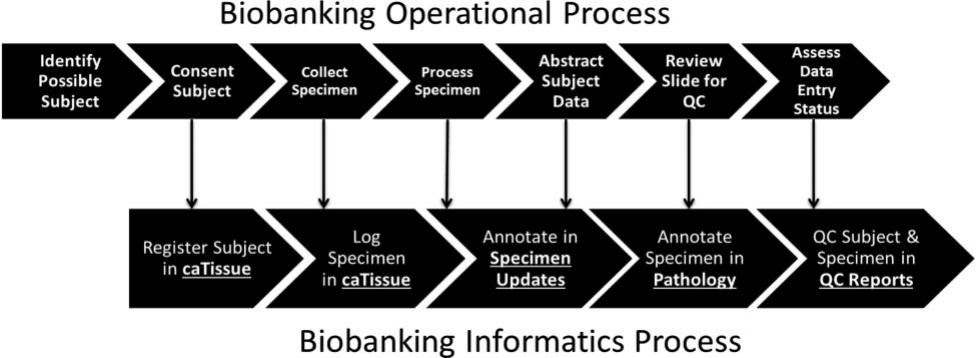
Biobanking operational process mapped to biobanking informatics processes supported in Acquire. Specific Acquire modules supporting operational processes are highlighted in bold, underlined text

Figure 1 maps the general biobanking operational activities in these workflows to Acquire modules for data collection.

Because many biobanks collect PHI such as participant name, date of birth and medical record number (MRN), secure authentication and authorization are critical components for almost any biobanking informatics system. Biobanks are often housed within a single institution that also acts as a coordinating center, though some have data entry staff at affiliate organizations. Acquire accommodates these two scenarios through a sophisticated single sign-on mechanism that is able to first query the data coordinating center’s AD/LDAP and then Acquire’s local database. Staff from the data coordinating center therefore can use their organizational username and password, whereas those at affiliate organizations use custom login information.

Authorized end users may login to the system or request support via a customized CAS login screen. Information on how to add new institutional logos and other branding is present within the Deployment Guide. Successful logins result in the generation of a session-based token that allows single sign-on to all modules of the system. Because of this token, it is critical that end users log out when not actively using the application. As an added security feature the system logs end users out after periods of inactivity spanning 5 min by default. From the login page, new users can request accounts, and these requests are reviewed by Acquire administrators.

### 3.1 Dashboard homepage

**Fig. 2.**
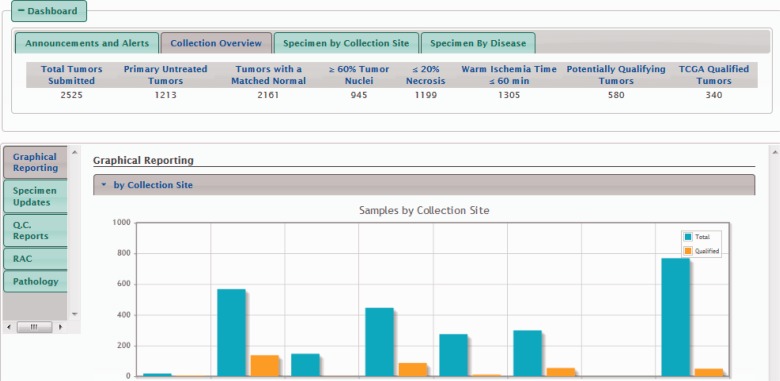
Acquire Dashboard and QuickViews. The Dashboard (center), Quickviews (center left vertical tabs and right center view) are displayed. Other modules are not shown, but are present at the top of the Acquire internal home page above the Acquire banner and Dashboard

Biobanks can be structured as geographically distinct collection and repository sites functioning as a single unit (i.e. a federated biobank), two or more collection sites with one central repository site or a standalone biobank. Regardless of structure, biobanks can support multiple protocols from different initiatives, meaning that the same staff can have different roles and use more than one standard operating procedure (SOP). Acquire supports all of these diverse structures or collection programs. Viewing permissions for each end user are managed through the Admin console, where distinct privileges can be assigned by each program and collection or repository site. All end users, however, can view data in the Dashboard from their programs (Fig. 2).

#### 3.1.1 Announcements and alerts

Within the Dashboard section, the default selected tab is Announcements, which shows alerts, news and other items that will be shared with all users within a program. Announcements are added by Acquire Administrators using a Microsoft Word-like graphical user interface console in which special characters, colored font or background and other modern standard word processing features may be utilized. Administrators can access the console, create new messages, manage existing messages and specify message expiration dates at which time the message will automatically be removed from the Announcements view from the Announcements dashboard.

#### 3.1.2 Collection overview

This tab contains a text-based summary health report for the biobank, where sums of collected cancer specimens and matched normal are provided. For example, a major user group of Acquire, the Texas Cancer Research Biobank (http://txcrb.org), had a collection protocol in which tumor specimens and matched normals were harvested and immediately shipped to the sequencing facilities within the Human Genome Sequencing Center at Baylor College of Medicine or were processed for shipment to The Cancer Genome Atlas (TCGA). Because this and other biobanks generally adopted TCGA standards for using key data as a proxy for specimen quality with regard to relative quality of the specimens for genomic and transcriptomic experimentation, these standards are shown within the Overview. These data include warm ischemia time below 60 min, percent necrosis and cellularity. Matched tumor and normal pairs meeting these TCGA-like criteria are tallied. Specimen pairs missing some key criteria (e.g. warm ischemia time not yet recorded, but necrosis and cellularity data available), but for all available data within acceptable parameters are tallied as potentially meeting TCGA-like criteria.

#### 3.1.3 Leaderboards

The Dashboard also contains two tabs for Specimen by Collection Site and Specimen by Disease. The Collection Site tab provides overview reports for each of the program’s biobank specimen collection sites and any collection subsites. For each parent and child collection site, the tallies of matched tumor/normal pairs for the data elements within the Collection Overview are shown for that particular site along with the total collections in the last 1 and 2 weeks. The temporal data were added to measure each site’s collection progress and help identify possible operational issues before they become major bottlenecks for the biobank. The Disease Site tab’s reports break the Overview data down similarly, but unlike the Collection Site tab, the Overview data elements are grouped by ICD-O-3 anatomic axis values, such as breast or brain.

### 3.2 QuickViews

Underneath the summary Dashboard, end users may view data and reports within one of several QuickViews, each of which provide more tailored information than the Dashboard. These views, shown as vertical tabs in Figure 2, are shown only for end users with appropriate permissions and, with the exception of summary Graphical Reports, the data displayed within each QuickView are limited to the biobank sites to which the end user has privileges.

#### 3.2.1 Graphical reports

Frequently investigators require graphs for presentations or grants. Graphical reports summarize the Dashboard reports for specimens collected by disease type, specimens collected at each biobank site and the rate of collection for the program for milestone tracking.

#### 3.2.2 **Specimen updates: workflow management**

Well-annotated biospecimens must be collected and processed as part of a complex workflow that spans different roles such as a principal investigator (PI), consent nurse, pathologist or their proxy, biobank supervisor and biobank technician. Specimen Update QuickViews help PIs and other biobank staff identify at what step in the process a specimen is by displaying status flags of procedural steps that are still unmet to help ensure efficient operations. Status flags include Awaiting Path, warm ischemia time, Prior Tx (treatment), NA (nucleic acids) Lab Qualified and whether or not the specimens were shipped to one of several types of facilities. To remove these flags, end users can click specimen (labels and barcode) and participant data in any row to automatically open up that record in Acquire’s caTissue Suite and perform the necessary data entry steps.

#### 3.2.3 QC and assurance reporting

A biobank must be able to assure its users that the specimens within the bank are correctly categorized and of good quality for downstream experimentation. Without mechanisms for QC/QA, tens of thousands of dollars can be spent to collect specimens of limited utility to scientific research and assay validation. A series of reports were created that display key quality metrics about specimens in the repository including the collection site or subsite, disease diagnosis (ICD-O-3 for these cancer specimens), anatomic site and initial quantity of tumor.

For the All TCGA Qualified Specimen report, these metrics include data on specimens that meet the TCGA requirements of percent necrosis, percent tumor nuclei and warm ischemia time. Potentially Qualifying reports show specimens that may meet the full TCGA criteria but are missing at least one required value. Molecular quality reports include RNA integrity number (RIN) values for RNA quality measurements and gel images for establishing DNA quality. Each record in this report is linked to the parent specimen. The parent specimens are annotated with warm ischemia time which, when reviewed in conjunction with RNA and DNA quality metrics, can assist a lab manager in determining whether the standards for specimen quality are being met. For these and other reports, end users can view additional details on the participant by clicking on any ‘MRN’ record. ‘MRNs’ can be any unique patient identifier, and these data are linked to the appropriate participant page in caTissue. For most reports, end users may select among biobank sites to which they have viewing privileges. For all reports, one can sort all columns, filter data in any column by entering text into the Google-like search fields located within the heading of each column and export reports into Excel.

QC reports by Site contain details about the specimen quality such as percent necrosis, whether the patient received prior treatment, specimen type, amount of available specimen, etc. Each record in this report links back to the specimen or participant detail page within caTissue. Perhaps most importantly for biobanks integrated tightly with genomics facilities, there is a flag to indicate whether each specimen has been shipped to an ‘omics core facility. The second report, NA Lab Quality Results by Site, provides information to biobankers on the quantity and relative quality of extracted DNA or RNA with links to caTissue as before.

#### 3.2.4 Pathology

Though caTissue has the capacity to dynamically add new data elements and forms, these data are stored in a manner that renders them difficult to extract and exchange in an interoperating system. Some pathologists or pathologists’ assistants prefer to directly enter data into Acquire, whereas others utilize biobank staff as their data entry proxies. The Pathology QuickView satisfies both scenarios by allowing end users with pathology privileges to add staging and grading data, information on percent cellularity and necrosis of the specimen and pathology reports.

#### 3.2.5 Resource allocation committee

**Fig. 3.**
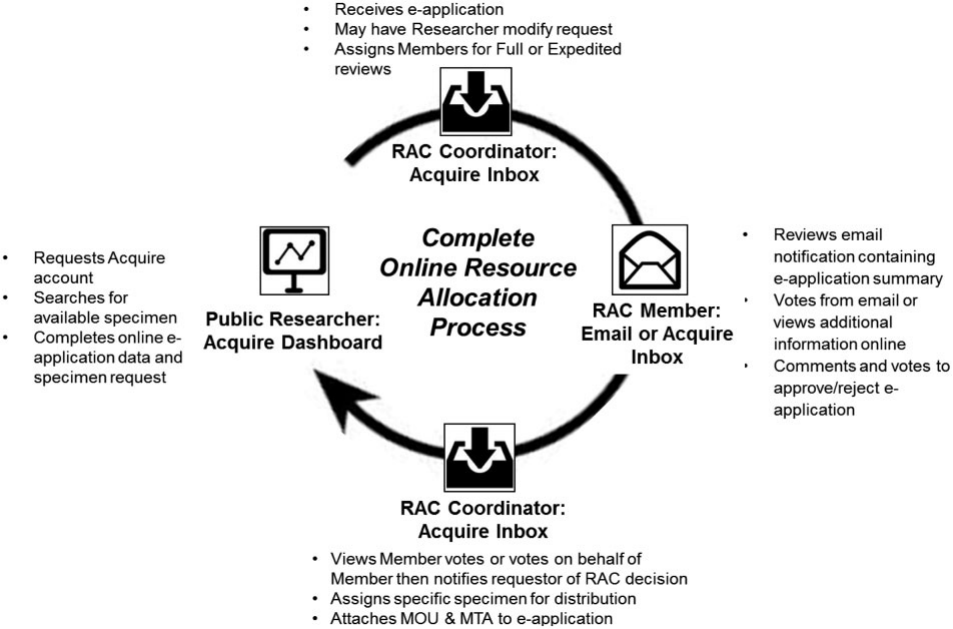
Acquire’s workflow management of resource allocation to public researchers. The Public Researcher executes a Specimen Availability search in their Acquire dashboard to determine if suitable specimens exist within the system (at left). The Public Researcher must then fill out an electronic request form (at left), which are submitted for review by the RAC Coordinator (at top). The Coordinator can reject requests or assign them to RAC Members for either full or expedited reviews. Assigned RAC Members can electronically ‘discuss’ and vote on requests from their RAC console (at right). Alternatively, these comments and votes can be captured by responding to emails from Acquire. The RAC Coordinator will review votes and formally approve or reject requests (at bottom). For approved requests, the Coordinator attaches pertinent documentation such MOUs and MTAs. Researchers can create new requests at any time (at left)

Specimen and data distribution oversight generally is managed by a RAC consisting of experts in biobanking-related fields and often a patient advocate. Acquire provides full electronic support for public researchers to request specimens, for recording all documents relating to these requests and for demonstrating that the biobank is sharing tissues per its charter (Fig. 3).

Once a public researcher has submitted a request as described within Section 3.4, the RAC module notifies the RAC coordinator of the request via email. The coordinator can login to Acquire to view and manage the request in their RAC Inbox. The coordinator may amend the application as needed on behalf of the requestor to add pertinent information for the RAC review. Each application is assigned by the Coordinator either to the full RAC or a subset of RAC members as an expedited review. The RAC members assigned to the request receive an email from the system that includes a summary of the application and a link to comment or vote on the request in Acquire. In some cases, RAC members have informally discussed requests with collaborators ahead of time and essentially provided informal pre-approval to those parties. The ability to vote directly via email supports rapid processing of this type of Expedited request. For other Expedited or Full RAC review requests, RAC members can respond to automated emails with questions and comments, which are injected into the request record at the bottom of the application in Acquire. If the RAC approves the request application, the status is updated to Approved, and the coordinator can select specimens to distribute per that biobanks SOPs. Utilizing the distribution protocols in the caTissue module, biobank site/s staff is notified of what must be distributed and to whom. The application is then removed from the RAC Inbox and placed in the RAC Archive for future reporting. If the RAC rejects the application, its status is updated to Rejected and moved to the Archive.

### 3.3 Data miner

Any member of the biobank, regardless of their role, can search their program’s biobank data (excluding PHI) via the Data Miner module. Members can specify one or more values for a variety of search parameters spanning participant, tumor specimen and matched normal data elements. The miner reports the number of matching specimens and allows end users to view and download the retrieved results or to modify their query.

### 3.4 Specimen availability and access requests

For public researchers to request specimens and data, they must self-register for public user account from the Acquire login page. Requests are reviewed by the Acquire administrator. Approved public users receive an automated email notifying them that an account has been created on their behalf. Upon login, public researchers can access only a limited public version of Data Miner, which contains a small subset of the query data elements within the Miner tool. This specimen availability tool retrieves the total number of specimens matching the search criteria so that requesters can determine if they want to submit a formal electronic application for specimen and data access. The data elements included within this query interface were selected to provide an overview of available specimens in the biobank while respecting stakeholders’ rights to protect the intellectual capital within the data.

If the requester elects to submit an online application, the form records their contact information; detailed project information including funding agency, award number and Institutional Review Board (IRB) approval; detailed specimen criteria; requested material type criteria such as frozen tissues, formalin-fixed paraffin-embedded slides, DNA, etc.; declarations of any financial conflicts of interest; and electronic certification of the acceptance of the biobank’s policy and procedures. Requesters can save incomplete forms, upload supporting documents and submit completed forms. Once submitted, they can track the application’s RAC review status, upload supporting documentation and view prior requests.

### 3.5 caTissue suite

This application and its management of core participant demographics; specimen collection, accession, management and distribution; and capacity for adding additional data elements as ‘dynamic extensions’ is described in detail within its online user documentation and training modules, http://1.usa.gov/1GHE9Qi. For Acquire, the software has had patches and updates applied to correct browser incompatibility issues with Firefox and to include additional bulk operation templates. Acquire not only displays hyperlinks to specimen and subject pages within caTissue from its Dashboard and QuickViews, but also has a hook to caTissue from study sites. For the reports and links to function properly, the collection site values within caTissue must match those within Acquire’s reporting modules.

### 3.6 Shipping forms

Acquire generates summary forms to accompany biospecimens that are shipped to genomics, PDX and cell line creation facilities. Biobank staff can add specimen to a shipping form, which is saved within Acquire. Upon completion, the specimens’ available quantity is updated and flags are set to indicate that the specimens have been distributed. Forms can be printed for inclusion in shipment containers and/or electronically sent to the core facility. Shipment forms in Acquire are based upon XML templates, and these were tailored for shipping specimens or nucleic acid derivatives to a genomic sequencing facility and tissue microarray creation core. By updating the XML template found within the Acquire codebase on GitHub, one can create new Shipping Forms.

## 4 Discussion

### 4.1 Utilization of Acquire for biobanking processes by role

Biobank operational processes (Fig. 1) begin with a consent staff person meeting with potential participants to obtain informed consent. Participant demographic and consent data are then entered into the caTissue module (Section 3.5). When specimens are collected, they are delivered by consent staff to biobanking technicians for accessioning and processing. The consent staff or biobank supervisor may later receive a pathology report and enter key abstracted pathology data in the Pathology module (Section 3.2.4). Because there may be a gap of several weeks between initial consent and receipt of additional information from the hospital or clinic, this staff member can take advantage of Acquire’s Specimen Updates module (Section 3.2.2) to quickly search for a participant’s record and follow a link to the correct data entry page in Acquire.

As biobanking technicians receive specimens from consent staff, they log specimen accession information, any biobank identifiers and the storage location in the caTissue module (Section 3.5). Technicians may create aliquots or derive new types of specimens such as DNA or RNA from the ‘parent’ specimens. Each aliquot and derivative data can be hand entered or, when processing larger numbers of these samples, imported in batch via bulk operations within caTissue.

Some protocols generate genomic, transcriptomic or other ‘omics data from collected specimens. Biobank technicians or supervisors can select specimens for distribution to an associated core facility within the Quality Assurance reports module (Section 3.2.3) and add these specimens to a Shipping Form (Section 3.6). When core facility staff receives the specimens, they can compare barcodes and labels to the expected data based in the form’s manifest to confirm the identity of each specimen. As DNA or RNA derivatives are created for ‘omics experimentation, quality metrics and other data are added within Specimen Updates by core staff (Section 3.2.2).

PIs frequently serve on RACs (Fig. 3), but they also must oversee the collection progress of their biobank(s). These individuals can utilize the reports within the Dashboard (Section 3.1) to monitor the overall ‘health’ of the bank.

### 4.2 Acquire’s impacts on biobank value

The biobanking field has struggled to incorporate additional mechanisms to improve the quality and utility of collected specimens. Acquire adds value by providing a mechanism through which the collection site can monitor molecular quality metrics DNA quality and RNA RIN numbers alongside specimen quality metrics such as percent cellularity, warm ischemia time and images of slides that underwent pathological review. Poor quality metrics often point to a need to adjust SOPs or to ensure that staff is accurately following protocol. These integrated data therefore allow biobank staff and leadership to identify areas in need of improvement. Within the Texas Cancer Research Biobank (TCRB) for example, the NA QA/QC report identified a group whose collections had consistently poor RIN values due to high warm ischemia time. TCRB leaders modified the method of collection in a way that did not alter standard of care, but dramatically improved RNA quality.

Lack of discoverability of specimens represents a serious issue within biobanking, leading to low numbers of distributions. Collection of tissues that are unused is neither fiscally practical nor does it maximize participants’ contributions to science through their specimen donation. Acquire’s public researcher interface facilitates specimen discoverability by public scientists and provides a streamlined method of applying for those specimens online.

Both of these features and others set Acquire apart from other commercial and open-source biobanking informatics solutions. Table 1 summarizes current key features of Acquire including caTissue and all other modules relative to other popular biobanking products. We annually review biobanking products to determine whether lower cost, complete solutions exist. First, we define a set of requirements based on our biobanks’ needs, and then we review the ISBER MarketPlace for inventory solutions, speak with colleagues and identify products at conferences. We then review product documentation to determine ‘best of breed’ based on our requirements. For those, we speak with the vendor and if possible current customers, and view product demonstrations. Further, we estimate the total cost of ownership based upon our total number of end users, active protocols and support needs. For commercial products, licensing models and costs can differ dramatically.

**Table 1.** Comparison of features of Acquire and three biobanking systems

System feature	Acquire	caTissue stand alone	BioFortis	Freezer-works
Supports biobank programs and virtual banks	Yes	Yes	Yes	No
Freezer and container management	Yes	Yes	Yes	Yes
Participant consent and study registration	Yes	Yes	Yes	No
Flexible creation of work flows based on IRB protocols	Yes	Yes	Yes	No
Cancer molecular and pathological QC metrics	Yes	No	No	No
Comprehensive reporting and dashboards	Yes	Limited	Yes	Limited
Billing module	No	No	No	Yes
Participant annotation	Yes	Limited	Yes	No
Comprehensive clinical annotations	Yes - next release	No	Yes	No
Specimen annotations	Yes	Yes	Yes	Yes
Molecular annotations	Yes	No	Yes	No
Public researcher requests for specimens	Yes	Limited	No	No
Tracking of RAC reviews	Yes	No	No	No
Clinical trials management system interoperation	Yes – bulk operations	Yes – bulk operations	Yes	No

In our last annual review, we started with a list of two open-source and seven commercial products. Initial documentation reviews compared with our requirements narrowed this list to two open source (caTissue and OpenSpecimen) and three commercial (BioFortis, TissueMetrix and LabVantage) products that might best fit our needs. Perhaps not surprisingly, the three commercial products shared a substantial number of feature sets. Ultimately, our analysis showed that in our environment with hundreds of active end users, hundreds of thousands of biospecimens and dozens of active collection protocols, Acquire is the best overall option.

The products in Table 1 were selected as examples of open-source software (caTissue), lower cost commercial general banking software that had few features meeting our requirements (Freezerworks) and higher cost commercial software better tailored to cancer biobanks (BioFortis) we reviewed. Summary data for caTissue can be generally applied to OpenSpecimen and data for BioFortis to other cancer commercial products. The three systems represent the full spectrum of licensing costs, though licensing models and features for any product can change with time. Our annual review process is useful for any biobanking informatics group, but due to potential differences in institutional requirements, factors contributing to license cost (e.g. number of end users, specimen or protocols) and budget, different products will be more or less attractive to different organizations.

### 4.3 Utilization of Acquire’s models

Each Acquire module was created based upon input from personnel representing at least three different biobanking programs, some of which themselves were federated biobanks with diversity in process at individual sites. These models are general enough to be useful to most human cancer biobanks, though the utility of most of the workflows and much of the technical models is not limited to human tissues. Rather they can also be of use to any specimen collection effort. Software development groups are free to utilize the models and workflows for Acquire and its modules. UML models were compared with the more abstract Clinical Data Interchange Standards Institute’s BRIDG model (Fridsma *et al.*, 2008) and an important future goal will be to harmonize the full Acquire model with the domain model.

### 4.4 Minimal resources required to adopt acquire

The system is freely available under an LGPL license, but adoption or adaptation of any software program has some associated personnel and hardware requirements. Though one could run CAS, caTissue and Acquire on one machine, with virtualization, it is possible to maintain them separately. At least one other server is required for its database, per N-Tier best practices. At a minimum, an institution would need personnel to fill the following roles: Linux system administrator with at least 10% FTE; database administrator 5%; and a programmer/analyst at 25% FTE, with new custom enhancements, new features and other changes requiring higher levels of effort. Acquire’s main back-end database is Oracle 11g, though a programmer/analyst familiar with JPA can take advantage of its platform neutrality to substitute the database of choice. Ideally, the systems would be backed up nightly to a remote location and the database archived at least once a week, representing additional storage and IT support costs that vary by institution. If potential adoptees are not familiar with the technical workings of caTissue, it is recommended that they purchase an annual support contract from a support service provider.

### 4.5 Future directions

In the post-TCGA era, biobanks must exhibit value to participants and researchers, instead of solely focusing on quality (Simeon-Dubach and Watson, 2014). Those banks embedded within an ‘omics research context must be able to support the next generation of research questions. Already the system can support longitudinal studies and specimens whose quality does not match that of TCGA’s stringent criteria. Acquire will change its leaderboards to expand to include criteria such as molecular biomarkers or datasets, completed minimal clinical and pathological data entry and other key elements determined by our multistate user community. We are currently assessing interoperation with OpenSpecimen as a second inventory module option within Acquire. Other enhancements slated for the next 2 years of development include:

#### 4.5.1 Clinical and pathological enhancements

We are incorporating a Clinical Annotation module to capture rich clinical and pathological annotations. Out-of-the-box forms will include sociogeographic data, self and familial history of cancer, patient risk factors and follow-up—all of which are generally useful for any cancer. Some pertinent data, however, cannot be generalized across tumor types. Because breast cancer is a complex and fairly well-described set of diseases and one of the most common cancers, we built out detailed forms to capture pathologic data, treatment regimens/exposures, patient-level biomarkers (e.g. serum, germline mutations, etc.), and biospecimen molecular biomarkers as a pilot. The new Annotations module is undergoing internal user acceptance testing now and will be available in Acquire’s next release slated for Spring 2015. A subsequent release to enhance form administration component will follow. The intent with these annotations is not be to create an exhaustive collection of knowledge on the participants that recapitulates an electronic health record, but to select for information that might have the most research utility.

Many clinical research protocols collect participants’ specimens for correlative or other purposes. As participants are consented to trials and specimens collected at specified time points within a trial’s calendar, data can be entered into the CTMS, exported as an Excel report from the CTMS and imported via bulk operations in Acquire’s caTissue module. Different clinical trials management systems expose data to varying extents. Some, such as Forte’s OnCore, have a small application programming interface (API) for consuming data about subjects that have enrolled in trials. These services include demographics, a protocol identifier, associated study participant identifiers and other key information. An important goal for the next release will be to create an Acquire API that consumes OnCore’s service for protocols that have a collection component. OnCore is the highest prioritized CTMS because of its high adoption rates among cancer centers and reputation as being a ‘gold standard’ system within the community.

#### 4.5.2 Improved PDX and cell line support

Though it is possible to manage PDXs and cell lines within Acquire now, enhancing support for tracking passages of tumors/cells and the quality assurance data that are often collected every few passages is an important goal for Acquire.

## 5 Conclusions

Acquire is a secure, open-source, web-based database that serves as the hub of many biobanks and has broad enough support for general biobanking workflows that it can be adopted and adapted by other biobanks. Heavy emphasis is placed upon oversight of biobanking workflows and QC within the system. Fully electronic support of specimen request, review and distribution of specimens and data for approved requests is included. Links to broad, concisely written consents and materials transfer agreements developed by biobanks such as the Texas Cancer Research Biobank complement Acquire and are also freely available for use by other cancer biobanks.
